# Retinal and Brain Organoids: Bridging the Gap Between *in vivo* Physiology and *in vitro* Micro-Physiology for the Study of Alzheimer’s Diseases

**DOI:** 10.3389/fnins.2020.00655

**Published:** 2020-06-17

**Authors:** Carlo Brighi, Federica Cordella, Luigi Chiriatti, Alessandro Soloperto, Silvia Di Angelantonio

**Affiliations:** ^1^Center for Life Nanoscience, Istituto Italiano di Tecnologia, Rome, Italy; ^2^Department of Physiology and Pharmacology, Sapienza University of Rome, Rome, Italy

**Keywords:** induced pluripotent stem cell, Alzheimer’s disease, Neurodegeneration, 3D cell biology, brain organoid, retinal organoid, *in vitro* disease modeling

## Abstract

Recent progress in tissue engineering has led to increasingly complex approaches to investigate human neurodegenerative diseases *in vitro*, such as Alzheimer’s disease, aiming to provide more functional and physiological models for the study of their pathogenesis, and possibly the identification of novel diagnostic biomarkers and therapeutic targets. Induced pluripotent stem cell-derived cortical and retinal organoids represent a novel class of *in vitro* three-dimensional models capable to recapitulate with a high similarity the structure and the complexity of the native brain and retinal tissues, thus providing a framework for better mimicking in a dish the patient’s disease features. This review aims to discuss progress made over the years in the field of *in vitro* three-dimensional cell culture systems, and the benefits and disadvantages related to a possible application of organoids for the study of neurodegeneration associated with Alzheimer’s disease, providing a promising breakthrough toward a personalized medicine approach and the reduction in the use of humanized animal models.

## Introduction

Alzheimer’s disease (AD) is a progressive age-related neurodegenerative disorder characterized by cognitive and psychiatric symptoms, such as memory and cognitive impairments, behavioral abnormalities, disorientation, and circadian rhythms and sensorial disturbances (Lane et al., 2018). Although environmental factors have been implicated in AD (Killin et al., 2016), genetics plays a key role in disease’s pathogenesis. Two main forms of AD can be distinguished: the familial autosomal dominant form (fAD) and the sporadic counterpart (sAD); sAD is further divided into early onset (average onset age <65 yo) and late-onset (average onset age >65 yo). Mutations in three key genes associated with the amyloid metabolism, namely amyloid precursor protein, presenilin 1, and presenilin 2 are linked to fAD (Dorszewska et al., 2016). However, fAD accounts only for less than 1% of the cases (Ryman et al., 2014), and the majority of AD cases are sporadic, though a genetic susceptibility has been identified in those individuals carrying the apolipoprotein E gene (Verghese et al., 2011; Giles et al., 2017). While the main scientific hypothesis underlying Alzheimer’s pathogenesis is amyloid-driven (Hardy and Higgins, 1992; Hardy, 2002; Wang et al., 2017), it is still debated whether alterations in tau protein’s function are a consequence of an impaired amyloid metabolism or it may act by itself, or synergistically to amyloid protein, to trigger the pathology (Nisbet et al., 2015; Takeda et al., 2015).

Albeit the genetic factors and individual susceptibilities, the AD brain displays a spread tissue degeneration with dense extracellular deposits of toxic amyloid-β (Aβ) oligomers and hyperphosphorylated tau-enriched intracellular tangles. Mitochondrial dysfunction, diffuse reactive oxygen species, and neuroinflammatory response are, among other, tissue, and cellular manifestations (Deture and Dickson, 2019). Diagnostic methods to assess the presence of AD biomarkers relies on brain imaging techniques, such as computed tomography, magnetic resonance index, and positron emission tomography in combination with the analysis of cerebrospinal fluid with high cost and invasiveness (Khoury and Ghossoub, 2019). Additional non-invasive and less expensive approaches are also under validation to support an early diagnosis of AD. Recently, novel diagnostic approaches are focusing on the imaging of patients’ retina and eye microvasculature (Patton et al., 2005; Hadoux et al., 2019). The rationale for exploiting such tissue relies on the common embryological origin of the retina and the brain (Bambo et al., 2014; Chang et al., 2014; Javaid et al., 2016). Remarkably, anatomical and functional alterations, such as the thinning of the ganglion cell and retinal nerve fiber layers (Paquet et al., 2007; Thomson et al., 2015), the presence of protein aggregates, and the glial activation (Koronyo et al., 2017; den Haan et al., 2018; Grimaldi et al., 2018, 2019) can be detected in the post-mortem evaluation of retinae of AD patients and humanized AD animal models, thus strengthening the idea that the retina could be exploited in early AD diagnosis.

Moreover, visual deficits, including difficulty reading (Rinaldi et al., 2008), depth perception (Mendez et al., 1996), and color recognition (Della Sala et al., 2000), are also reported in the early stages of AD. All these efforts aim to find specific biomarkers and tools for AD diagnosis at earlier stages, possibly when synapses and neuronal functions are not yet compromised, allowing pharmacological and clinical interventions (Frisoni et al., 2017). In this context, understanding the biological basis of AD progression remains a major scientific challenge, largely because of the complexity of the human brain cell interactions. Indeed, the lack of appropriate preclinical models of the human neurodegenerative pathologies, and in particular AD, hinders the understanding of pathological mechanisms and consequently the development of effective and safe diagnostic procedures and therapies.

### From Humanized Mouse Models Toward Stem Cell-Based 3D *in vitro* Approaches

While more than 200 functional transgenic rodent models have been generated (Hall and Roberson, 2012), they only partially recapitulate pathogenic traits of AD, and to date, none of the potential drug candidates screened up to now has shown effective therapeutic outcomes in humans, making the translation into clinics of these drugs controversial (Laurijssens et al., 2013). Thus, the continuous development of novel *in vitro* disease models, which more comprehensively recapitulate hallmarks of AD, represents an important research strategy that may enable to accelerate the molecule screening process before testing a lead candidate *in vivo* on transgenic rodent AD models.

The identification of the key molecular factors (Sox2, Klf4, c-Myc, and Oct3/4) necessary for bringing back to stemness an adult somatic cell (the so-called “Yamanaka factors”) has posed the basis for a revolution in the field of *in vitro* human disease models (Liu et al., 2008). iPSCs represent a pluripotent stem cell class capable of differentiating, in response to specific small molecule stimuli, into each of the three embryonic germ layers and therefore, in principle, to any terminally differentiated cell type (Takahashi and Yamanaka, 2006). These cells exhibit features similar to those found in embryonic stem cells (ESCs) and can be potentially derived from different somatic cell types. Before iPSCs, the development of stem cell-based *in vitro* models relied mostly on mouse ESCs and in some limited cases on human ESCs, although, in the latter, the poor availability due to legal and ethical constraints has negatively affected their usage (Robertson, 2001). Thus, the use of these new stem cells has fostered the efficient generation of versatile human-based cellular models to study disease-relevant genome-specific alterations preventing the ethical concerns associated with the use of human ESCs.

The advancements in three dimensional (3D) culturing approaches (Baker and Chen, 2012; Duval et al., 2017) together with the regenerative potential of iPSCs have driven the generation of a plethora of new protocols for creating 3D micro-physiological systems *in vitro* to exploit as an alternative and complementary approach to humanized animal models for the study of species- and patient-specific features (Centeno et al., 2018).

Several newly designed 3D cell culture methods have been proposed, spanning from cell-laden 3D scaffolds (Park et al., 2018) to induced pluripotent stem cell (iPSC)-based cerebral organoids (Grenier et al., 2020) and 3D bioprinting (Salaris et al., 2019). Cell-laden structures can be realized by encapsulating neuronal cells within a variety of materials (Kratochvil et al., 2019; Madhusudanan et al., 2020), either natural and synthetic, that might replicate some pivotal features of the native extracellular matrix (ECM), including stiffness and ECM-dependent pathways (Dutta and Dutta, 2009). While the stiffness of plastic can hardly replicate the mechanical environment provided by the brain ECM, the use of biocompatible ECM-like gels (hydrogels) may provide several benefits (Badylak et al., 2009). Hydrogels, such as collagen, gelatin, hyaluronic acid and similar, provide a tissue-like water content, and tunable biochemical and mechanical properties, thus supporting the generation of tailored 3D microenvironments in which cells can self-organize in distinct tissue architectures (Burdick and Vunjak-Novakovic, 2009). Additionally, the biological stimuli coming from the surrounding 3D microenvironment contribute to the long-lasting survival, and functional maturation of cells typically challenging to be cultured *in vitro*, such as neurons. For instance, in 3D neural constructs, the increased dimensionality allows a more compliant physiological cell-cell interactions between neurons, thus limiting spatial overlap during neurite elongation and branching (Palazzolo et al., 2017). Moreover, several studies reported a less synchronous bursting activity and a relatively higher level of random spiking when comparing 3D neuronal cultures to conventional 2D cultures (Eytan and Marom, 2006; Severino et al., 2016). Frega and collaborators speculate that a wider cellular interaction within 3D cultures leads to a broad desynchronization of network activity, thus producing bursting activities only in specific neuronal subpopulations (Frega et al., 2014). In support of this hypothesis, locally synchronized electrophysiological patterns are also observed in other experimental models, such as acute rodent brain slices (Beggs and Plenz, 2003; Beggs, 2004), where are assumed to play an essential role in the maturation of developing functional circuitry. However, experimental observations in the field are still controversial and there is not a unified theory behind the nature and function of synchronous and asynchronous network activities.

Among this variety of 3D culture systems, a promising *in vitro* tissue-like 3D platform is represented by the brain and retinal organoids. Organoids are stem cell-derived cellular aggregates (Clevers, 2016), which, starting from a selected cluster of iPSCs, can acquire morphological and functional features as well as gene-expression patterns, similar to those found in the corresponding native structures (Lancaster and Huch, 2019). Indeed, since the first organoid protocol in 2009, a lot of effort has been made for generating organoids that mimic a wide range of human organ types, and nowadays an extensive scientific literature demonstrates their potentiality in modeling some human diseases (Rossi et al., 2018). Due to the greater diversity of cell composition and functions, organoids have the potential to create physiological cellular environments for modeling the biology of the human central nervous system (CNS) and might bridge the need for an efficient, reproducible and reliable *in vitro* 3D model for the conduction of preclinical high throughput drug screening directly on patient-derived tissue-like samples (Kadoshima et al., 2013; Lancaster and Knoblich, 2014).

### Alzheimer’s Disease in a Dish: Patient-Specific Brain and Retinal Organoids

**FIGURE 1:**
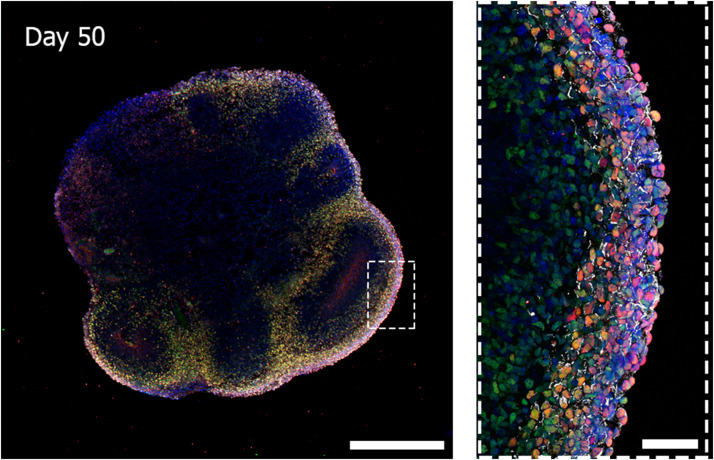
Three-dimensional biological complexity of a human iPSC-derived whole-brain organoid **(left)** and enlarged image of a representative cortical layer organization **(right)**. Immunostaining at day 50 of iPSC-derived brain organoid reveals the presence of a primitive cortical stratification containing early born deep-layer neurons (TBR1, red; CTIP2, green). Pan-neuronal dendrites structures are stained with MAP2 (white) and nuclei are stained with DAPI (blue). Scale bars = 500 microns (image on the **left**) and 50 microns (image on the **right**). Multiarea acquisition was performed with a laser scanning confocal microscope using a 60× magnification objective.

Cerebral organoids derived from human iPSCs can autonomously recapitulate the 3D architectural arrangement of the brain and spontaneously generate discrete brain regions including the dorsal forebrain, the hippocampus, and the retina (Figure 1).

Different methods to generate organoids have been already reported in the literature with different structural complexity and cellular diversity (Mariani et al., 2012; Paşca et al., 2015; Lancaster et al., 2017; Giandomenico et al., 2019). Among the established 3D organoids, two main categories can be identified: (i) self-patterned organoids, based only on the intrinsic capacity of stem cells to self-differentiate and assemble (Lancaster et al., 2013) in native-like structures, and (ii) patterned organoids, which exploits the use of small molecules to drive and lead the formation of specific brain regions (Mariani et al., 2012; Kadoshima et al., 2013; Paşca et al., 2015).

Lancaster and Knoblick first described the development of cerebral organoids from human iPSCs (Lancaster and Knoblich, 2014). Growing spherical iPSC aggregates in suspension, known as the embryonic bodies (EBs), enable the generation of radially organized neuroepithelial buds that mimics the cell-cell interactions observed during early embryogenesis and, supporting their growth through their embedding into ECM droplets, it is possible sustaining their apicobasal expansion. Over time, heterogeneously terminally differentiated cell types are generated and organized in well spatially defined structures that resemble human brain regions including dorsal forebrain layering.

Nevertheless, the generation of these cytoarchitectural structures relies mainly on the self-patterning and intrinsic mechanisms of stem cells, and the high variability, in terms of structure distribution, complexity, and maturation, remains one of the major limitations affecting this technology. This randomness influences the reproducibility of cellular responses to specific stimuli, thus generating a significant variance of collected data even on organoids belonging to the same iPSC line and compromising the wide exploitation of such models in clinical research (Lancaster et al., 2013). A recent work of Lancaster’s group demonstrated that combining self-patterned organoids with bioengineered microfilaments, such as poly(lactide-co-glycolide) copolymer (PLGA) fibers, the overall reproducibility of internal cytoarchitectures of the model can be improved, lowering the number of non-neural identities within the organoids (Lancaster et al., 2017). However, despite those improvements, the inter-organoids variability is not challenged, and it remains an important drawback.

On the other hand, the application of synthetic morphogens or exogenous small molecules, as in the case of patterned approaches, influences and tunes the iPSC fate to a specific range of neurogenic cell types, introducing specific morphogenetic constraints that result in a steady proportion of cell types generated within organoids and batches of differentiation (Quadrato et al., 2017; Sloan et al., 2017; Yoon et al., 2019). However, if from one side patterned organoids display a similar gene-expression diversity (Velasco et al., 2019), they fail to efficiently generate highly expanded cortical structures and thus may not represent the ideal platform for modeling morphological and structural changes that occur in neurological disorders typical of the adult brain as neurodegenerative diseases (Kelava and Lancaster, 2016).

Thanks to these remarkable features, brain organoids are finding a wide range of applications for the study of diseases affecting the human brain development, such as congenital brain malformation and neurological disorders, including age-related neurodegenerative diseases (Amin and Paşca, 2018; Papaspyropoulos et al., 2020). Recently, the use of iPSC-derived cerebral organoids led to replicate *in vitro* some molecular determinants of Alzheimer’s disease, such as the Aβ and tau pathology and the subsequent synapses dysfunction (Gonzalez et al., 2018). Independent studies reported that treatment with γ-secretase or β-secretase inhibitor compounds was able to partially inhibit the production of toxic Aβ and to reduce the hyperphosphorylation of tau proteins, suggesting the Aβ-driven tauopathy theory (Lee et al., 2016; Raja et al., 2016). Mook-Jung and collaborators reported the discovery of CDK-504 (Choi et al., 2020), a selective histone-deacetylase 6 inhibitor, which dramatically enhances the proteasome degradation pathway of pathological tau in AD patient-derived brain organoids and rescues synaptic deficits. The use of brain organoids can also accelerate Aβ accumulation in culture, thus facilitating the characterization of the associated cellular and molecular events (Raja et al., 2016). Indeed, the presence of a 3D matrix surrounding the cells may constrain the diffusion of pathologic proteins in the culture media, allowing their distribution in confined areas, and resulting in local protein aggregation and accumulation (Choi et al., 2014; Park et al., 2018). For instance, a consistent increase of Aβ42 fragment secretion and Aβ42/Aβ40 ratio, similar to pathological phenotypes observed in transgenic animal models, has been found in AD cerebral organoids (Pavoni et al., 2018). Moreover, co-cultures of regionalized organoids (Bagley et al., 2017; Birey et al., 2017; Xiang et al., 2017), which resemble distinct areas of the brain (i.e., dorsal forebrain, ventral forebrain, hippocampus), would allow the modeling of important aspects of human brain regions interaction, which might be exploited to investigate the spreading of toxic protein aggregates in the whole CNS.

Interestingly, as part of the CNS, the retina has demonstrated some pathological processes that occur in the brain during neurodegeneration. In AD patients, relevant visual deficits (Ngolab et al., 2019) sometimes appear before the first neurological symptoms, making the retina a potential *in vivo* tool to use for monitoring the onset and progression of the AD neurodegeneration (Liao et al., 2018). Therefore, the development of *in vitro* engineered tissue-like constructs, such as retinal organoids, that better approach the retinal tissue and physiology undergoing AD pathogenesis is reasonably also under evaluation (Artero Castro et al., 2019). As for brain organoids, independent pioneering works reported the *in vitro* generation of eye structures, such as retina (Capowski et al., 2019), retinal pigmented epithelium (Reichman et al., 2017), lens (Murphy et al., 2018), and cornea (Foster et al., 2017). Sasai’s work demonstrated that the formation of the optic cup-like structure could be achieved from mouse and human ESCs *in vitro* (Eiraku et al., 2011; Nakano et al., 2012). Similarly to whole-brain organoids, culturing iPSC-derived EBs in predetermined conditions generates 3D optic cup-like structures together with stratified neural retinal cell subtypes and progenitors. After this seminal work, several protocols have been proposed to drive the differentiation of human iPSC toward retinal organoids (Mellough et al., 2019). Current retinal differentiation protocols can produce most relevant retinal cell types in laminated fashion with a variable efficiency in generating functional photoreceptors (Zhong et al., 2014) to retinal ganglion cells (Ohlemacher et al., 2016), and overall mature retinal tissues. Indeed, retinal organoids do not routinely exhibit a complete maturation of the tissue with morphological or electrophysiological features characteristic of the adult retina *in vivo*. Several studies have successfully proven the utility of such *in vitro* models in some retinal degenerative diseases, such as glaucoma (Ohlemacher et al., 2016) and retinitis pigmentosa (RP; Gao et al., 2020). Retinal organoids have been also exploited as a tool to investigate retinal ganglion cell’s (RGCs) physiology, as a means to assess RGC’s regeneration potential and neuroprotective effect of new therapeutics (Fligor et al., 2018). However, while patient-specific retinal organoids represent a functional model for evaluating biological processes underlying the eye development and disease *in vivo*, such models are still not exploited in studies of neurodegenerative diseases of the CNS, such as in AD.

### Future Directions of the Organoid Technology

**FIGURE 2:**
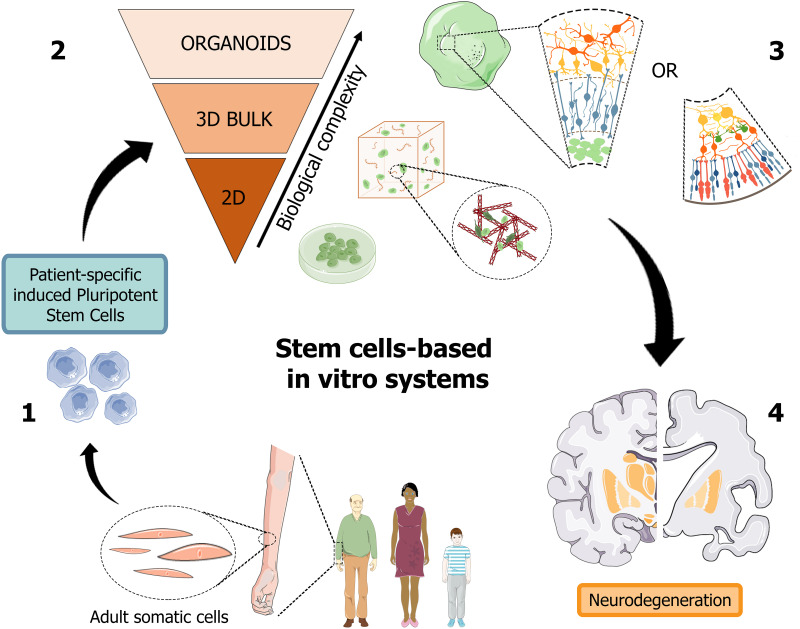
Schematic representation of human stem cell-based *in vitro* systems for neurodegenerative diseases. The iPSC building blocks (1) are obtained from cell reprogramming of somatic cells from patients or healthy donors. iPSC-derived neuronal cultures, either in the form of 2D and 3D cell cultures (2), are generated to reproduce the biological complexity of human tissues *in vitro*. Cerebral and retinal organoids (3) can reproduce *in vitro* cortical and retinal features, with all their structural and functional components, thus becoming promising patient-specific platforms for the study of neurodegenerative pathologies, such as Alzheimer’s disease (4). Figure 2 was assembled using images downloaded from Servier Medical Art, licensed under a Creative Common Attribution 3.0 Generic License. http://smart.servier.com/.

Recently, organoids opened an avenue to generate a versatile scaled-down version of brain and retina tissues, which may significantly help the study of the pathogenic mechanisms hiding behind neurodegenerative disorders, such as in AD, and foster the design and high-throughput screening of candidate molecules toward the development of a personalized diagnostic and therapeutic approach (Figure 2).

The use of patient-derived iPSC-based retinal and brain organoids together with advanced gene-editing techniques might provide a simplified sight into the mechanisms underlying AD-related alterations, predicting more comprehensive clinical outcomes and possibly overcoming some limitations imposed by humanized animal models. Importantly, preserving the genetic and epigenetic patient-specific background may provide a striking strategy for understanding such neurodegenerative mechanisms and constitutes an opportunity to identify pathology-related hallmarks not otherwise recognizable during medical imaging evaluation or post-mortem tissue observations (Gerakis and Hetz, 2019).

Nonetheless, the iPSC-based 3D organoid system is yet in its infancy, and, up to now, it appears to be not devoid of disadvantages. Although iPSC technology could serve as a promising platform for disease modeling, their application in translational medicine remains poorly diffused, due to a not negligible donor-to-donor variability and concerns on the impact of virus-mediated reprogramming protocols when these cells are proposed for *in vivo* transplantation. Besides, iPSCs may display an increased genomic instability, carrying tumorigenic loci (Hussein et al., 2011; Liang and Zhang, 2013) and retaining epigenetic memory belonging to previous somatic fate (Puri and Nagy, 2012).

A major issue for the use of such *in vitro* models in age-related neurodegenerative diseases is the optimization and validation of organoid’s generation methods enabling a precise, reproducible, and relatively fast maturation of the organoid. Indeed, while aging drives numerous genetic alterations resulting in a continuously different cellular transcriptional profile (López-Otín et al., 2013), up to now organoids display an immature phenotype peculiar to the late fetal stage of the tissue (Camp et al., 2015). Unexpectedly, even though culturing retinal organoids for an extended period, they still present an incomplete maturation, thereby making it challenging to replicate aging-related phenotype in a dish. Different methods are under development to accelerate the maturation process in iPSC-derived neuronal cells (Miller et al., 2013). For instance, the application of chemical stressors into the culture media as hydrogen peroxide (Campos et al., 2014) and telomerase inhibitors (Vera et al., 2016) facilitates an aging-like phenotype in iPSC-derived neurons in 2D cell cultures. However, while it is reasonable to hypothesize to apply a similar strategy to accelerate the aging process in 3D retinal and brain organoids, it is not being exploited.

Furthermore, different cell types are involved in the AD pathogenesis, and neuron and synaptic loss are only the final events of a more complex picture. Unveiling the role of different brain and retinal cells (i.e., neurons, astrocytes, microglia, endothelial cells) may play a pivotal role in designing novel therapeutic approaches (Marton and Paşca, 2020). To this end, 3D retinal and brain organoids provide an outstanding opportunity to explore cellular and subcellular functions within *in vitro* models that closely recapitulate the native 3D configuration of the human neural tissue. Another major challenge is represented by the role played by neuroinflammation in the neurodegenerative process (Hemonnot et al., 2019) and how this could be carefully taken into account within *in vitro* AD models. Indeed, while microglia, the resident immune cells of the CNS, are recognized to strongly contribute to the AD onset and progression, they do not innately develop into brain and retinal organoids. Microglia deriving from the hematopoietic lineage, colonize brain tissue during embryonic development (Cunningham et al., 2013; Swinnen et al., 2013). Hence, their incorporation in brain and retinal organoids will be critical to dissect their contribution in the early stage of the disease and identify new and patient-specific therapeutic pathways (Song et al., 2019). Recently, the innate generation of microglia cells within whole-brain organoids has also been reported (Ormel et al., 2018), however, there is still little evidence regarding the reproducibility of the method and further characterization of this approach is required.

Similarly, vasculature and blood-retinal barrier (BRB) alterations are widely reported in AD retina and their investigation as possible diagnostic tools is under evaluation (Zlokovic, 2011; Cheung et al., 2014; Zhao et al., 2015). Retinal tau protein plays a key role in regulating axonal transport and signaling in the retina (Ho et al., 2012). Reduced clearance of retinal Aβ and other neurotoxic substances contribute to BRB dysfunction and breakdown (Dinet et al., 2012), inducing a persistent inflammatory state (Wang et al., 2009). Further, the BRB, similarly to the blood-brain barrier, can act as a checkpoint to the transit of many drugs, and for these reasons, *in vitro* vascularization of retinal organoids using endothelial cells might contribute to foster the identification and development of new molecular targets (Achberger et al., 2019).

In conclusion, although there are yet several gaps to be filled before allowing the use of iPSC-based organoid technology in neurodegenerative and in translational studies, these *in vitro* platforms offer promising outlooks that might pave and foster the rapid growth of novel *in vitro* approaches to tackle AD (Park et al., 2019). Moreover, alterations in retinal structure and function have been reported in other neurodegenerative disorders, such as Parkinson’s Disease (Archibald et al., 2009). Thus, elucidating the neurodegenerative mechanisms that underlie retinal impairments may provide not only useful insights regarding the brain disorders’ onset and progression but also promising non-invasive tools for large-scale screening and monitoring brain diseases.

## Author Contributions

CB and FC wrote the manuscript with support from LC. AS took the lead in editing the manuscript. AS and SD supervised the writing and conceived the idea. All authors provided critical feedback and helped to shape the manuscript.

## Conflict of Interest

The authors declare that the research was conducted in the absence of any commercial or financial relationships that could be construed as a potential conflict of interest.
